# Structural and Intermediary Social Determinants of Health and the Emotional and Behavioral Health of US Children

**DOI:** 10.3390/children10071100

**Published:** 2023-06-22

**Authors:** Ngozi V. Enelamah, Margaret Lombe, Mansoo Yu, Melissa L. Villodas, Andrew Foell, Chrisann Newransky, Lisa C. Smith, Von Nebbitt

**Affiliations:** 1 Department of Social Work, College of Health and Human Services, University of New Hampshire, Durham, NH 03824, USA; 2 School of Social Work, Boston University, Boston, MA 02215, USA; 3 School of Social Work, Department of Public Health, College of Health Sciences, University of Missouri, Columbia, MO 65211, USA; 4 Department of Social Work, College of Public Health, George Mason University, Fairfax, VA 22030, USA; 5 Jane Addams College of Social Work, University of Illinois, Chicago, IL 60612, USA; 6 School of Social Work, Adelphi University, Garden City, NY 11530, USA; 7 The Grace Abbott School of Social Work, University of Nebraska—Omaha, Omaha, NE 68198, USA

**Keywords:** social determinants of health, child emotional and behavioral problems, mental health, mental health specialist visit

## Abstract

Children grow up in homes where varying environmental and socioeconomic contexts have a bearing on their emotional and behavioral health (EBH). This study used data from a representative sample of the child supplement of the US National Health Information Survey (NHIS) and applied the social determinants of health (SDoH) framework to explore factors associated with child EBH. We conducted a path analysis of the child’s EBH measured by the strengths and difficulties questionnaire (SDQ) from their macro and socioeconomic contexts, e.g., policy, household, and other health system risk factors. For children in the sample, aged 4 to 17 years old (n = 9205), most path relationships to child SDQ scores were statistically significant. The total effects from a child’s visit to a mental health specialist (0.28) and child’s age (0.22) had the highest coefficients to child SDQ scores. A modified model showed a better fit with X2 (4) = 22.124, RMSEA = 0.021, and 90% CI [0.013–0.03], CFI = 0.98. Findings indicate that child factors such as being older, the use of mental healthcare services, and family socioeconomic status were significantly associated with EBH, calling attention to the need for more responsive policy and behavioral health interventions that address household/familial and child-level factors, critical determinants of child wellbeing.

## 1. Introduction

Ecological factors associated with a child’s mental health, as well as emotional and behavioral problems, remain largely unexplored. Research on child mental health overwhelmingly concerns itself with intrapsychic and biological aspects [1], limiting the identification and adoption of more effective interventions. Few available studies [2] note the interconnected pathways and associations between different social contexts that interplay to affect child emotional and behavioral health (EBH) outcomes. Comprehensive frameworks such as the social determinants of health (SDoH) [3,4,5] connect psycho- and eco-social theories associated with an individual’s ecology to predict equity in health and may provide utility in modeling interrelationships between factors that affect a child’s mental health outcomes.

This study used representative data from the child supplement of the United States (US) National Health Information Survey (NHIS) [6] and the SDoH framework [5] to model the association of various risk factors and the EBH of children aged 4–17 years old. The study provided an overview of child mental health, focusing on emotional and behavioral aspects, and explored the paths of association between the macro (policy/societal), family, and child factors, to child mental health outcomes.

### 1.1. Child Emotional and Behavioral Health (EBH)

Emotional and behavioral health (EBH) or mental health problems in children are major challenges in typical learning, behavioral or emotional functioning. Most EBH problems disrupt normative development in a child’s learning, behavioral, and emotional processes [7]. These deviations from the norm are often masked as the child grows older and may continue to adversely influence outcomes later in the life course [8]. The most prevalent disorder among US school-aged children in 2021 was Attention Deficit/Hyperactivity Disorder (ADHD), affecting 6.0 million children (9.8%), followed by anxiety (9.4%), behavioral or conduct problems (8.9%), and depression (4.4%) [7,9]. Other common mental health disorders affecting children include social phobia, separation anxiety, oppositional defiant disorder, and autism [7]. Furthermore, mental health disorders among U.S. children have increased from 13% in 2013 to over 20% in 2015 [9,10]. For example, the CDC reported that one in six children between 2 and 8 years old (17.4%) in the US is diagnosed with a mental, behavioral, or developmental disorder annually [11,12], but fewer (80% of those that need help) access any treatment or services [9]. The state of a child’s EBH or mental health has implications for their long-term cognitive, social, and physical development [7,12]. EBH problems compromise the welfare of children, putting them at risk for lower math and reading scores and overall lower educational attainments [2], and contribute to consequences such as school dropout and discipline problems. Previous research suggests that many childhood EBH problems are associated with adverse future physical and mental health outcomes in later life [9]. For instance, past studies note that the age of onset (AOO) for disorders such as ADHD (7–9 years), oppositional-defiant disorder (7–15 years), or conduct disorder (9–14 years) is associated with a projected lifetime risk [13]. Indeed, child EBH disorders are a serious public health problem; costing over 10.9 billion USD annually [14], and coupled with this is the disadvantage of limited mental health service providers [9].

Moreover, emotional and behavioral problems are not often recognized as disorders early in life, as they are often clouded and viewed as normal growing distresses [2]. Children with EBH problems are at risk of substance use disorders and suicide [10] and heightened school disciplinary action [15]. More so, Black children and Hispanic children with emotional and behavioral disorders and ADHD (or emotional disturbance) are more likely to be at risk of disciplinary exclusion from school [15,16]. Beyond immediate challenges accruing to affected children, family stress, and national public health costs; other outcomes in adulthood such as chronic diseases, poor interpersonal relationships, underachievement, and more, trace their roots to childhood EBH problems [2,15,17,18].

### 1.2. Factors Associated with Child EBH Problems

Other underlying circumstances in the child’s life or from the parents, including stress and psychological distress in the mother’s life, have been indicated [19]. Specifically, research links increased cortisol during maternal experiences of stress in pregnancy to the fetus and suggest a linkage to EBH problems [19,20], including the heritability of child EBH problems [21,22,23]. In addition, residing in a home with poor health-seeking behaviors and having a low socioeconomic background are risk factors for EBH problems in children [24,25], especially for those aged between 6 and 9 years. Challenges such as ADHD may have a child exhibit hyperactive or disruptive behaviors that increase their presentation of more EBH problems [26]. Adverse or traumatic childhood experiences such as abuse or witnessing violence may also predispose children to mental health problems [27,28].

### 1.3. Social Determinants of Health (SDoH) Framework

There is limited literature examining the complex association between the factors in the policy, family, and social environments surrounding child EBH outcomes. None, to our knowledge, have used the SDoH framework to characterize the interplay of societal and family factors described as structural social determinants of health or delineated their relative influence on child EBH to support an evidence base for actionable steps. Social determinants or non-medical factors are inequities that affect health and are mostly changeable, normal conditions under which people are born, grow, live out their lives, and age, and which ultimately shape and affect their health outcomes [5,29,30,31]. The SDoH framework is based on the concept of ‘social position’ (an individual’s background and circumstances of life, stratifications, exposures, susceptibilities) and the social production of disease, and illustrates the influence of social inequalities on health outcomes [5]. The SDoH framing is important, as it has the potential to reveal how challenges at multiple levels such as family, household, and policy interact to impact child wellbeing.

### 1.4. Applying the SDoH to Child EBH

The current study narrowly draws upon the SDoH conceptualization as proposed by Solar and Irwin (2010) to model how a child’s contexts—socioeconomic and political—interact with his/her family’s position (structural determinants), and intermediary determinants such as material circumstances and biopsychosocial factors to influence the child’s mental health outcomes [5,32]. According to Solar and Irwin (2010), the primary output of the SDoH framework is health equity (which they posit governments have the onus of fostering). Health equity from the SDoH framework is defined as ‘the absence of unfair, avoidable, or remediable differences in health among social groups’ [29], and for this study, optimal EBH outcomes for children.

Contexts such as governance, macroeconomic policies, social and public policies, and culture/societal values are depicted as structural determinants of health or sociopolitical mechanisms that generate and maintain social hierarchies [5]. Structural factors (*stratifiers*), namely socioeconomic position, are influenced by education, occupation, and income. These *stratifiers* constitute social disadvantages that affect health. Drawing from the SDoH framework, this study sought to answer the following questions:

1. How do structural social determinants of health from the macro context, such as availability of welfare services, health insurance, and use of child well-checkups, affect a child’s EBH problems?
2. How are intermediary social determinants of health, such as household socioeconomic position (poverty level) and problems paying medical bills, associated with a child’s EBH problems?
3. What are the direct and indirect mediating effects of intermediary factors such as access to mental health services, food insecurity, and chronic disease on child EBH?

## 2. Materials and Methods

### 2.1. Data

This study used data from the sample child and family core modules of the 2016 Child Supplement of the US National Health Interview Survey (NHIS), a national and in-person household survey among the civilian non-institutionalized population conducted by the CDC and supplemented by the National Data Resource Center for CAHMI [6]. The child survey contains information on one focal child, 17 years old or younger (if any), randomly selected from each household and reported by the mother or caregiver. The results of the survey are adjusted and weighted for national representation in demographic composition. Unknown or missing values for this age group were approximately less than 0.3% and were not included in the analysis. This study focused solely on children aged 4–17 (n = 9205) who qualified for the assessment using the Strengths and Difficulties Questionnaire (SDQ) originally developed by Goodman [33].

### 2.2. Measures

Dependent variable. The child’s EBH problems were measured using an adapted version of the SDQ [33]. The original SDQ was developed as a five-factor 25-item screening tool to be completed by parents or teachers of 4–17-year-olds [33,34]. The survey used a modified 5-item SDQ version that represents five domains (conduct problems, emotional state, emotional symptoms, peer relationship problems, and hyperactivity/inattention) [20,35]. The 5-item version asked, “if the child, during the past six months was generally well behaved and usually does what adults request (conduct problems)”; “if the child had many worries, or often seems worried (emotional problems)”, “if the child was often unhappy, depressed, or tearful (emotional symptoms)”; “if the child gets along better with adults than with other children (peer relationship problems)”; and “if the child had good attention span, see chores or homework through to the end (hyperactivity/inattention)” [6]. Positive items generate *a difficulties* score, while the prosocial questions provide a *strengths* score. The sum of these scores generated the SDQ score, where higher scores indicate more significant difficulties or fewer strengths, or mental health problems.

*Validity and reliability of the SDQ instrument.* Our examination of the psychometric properties of the short 5-item SDQ instrument (details in another manuscript) used in this study yielded only four of five problem subscales upon orthogonal varimax and oblique Promax rotations, respectively, unlike the 25-item instrument reported to load onto a five-factor problems scale. While the SDQ measures loaded to three factors, namely conduct problems, emotional problems, and hyperactivity/conduct problems, an iterated principal axes (ipf) analysis upon varimax rotation yielded four factors that loaded to previously established subscales, namely emotional problems, conduct problems, hyperactivity/inattention, and peer problems, and confirmed by the loading plot. No variable measured the prosocial behavior domain. The reliability of the 5-item scale was a low 0.54, compared with the range of 0.67–0.83 found in most studies using the 25-item scale. The average inter-item covariance of the 5-item SDQ was 0.063, out of range of the optimum range of 0.20 and 0.40. As we note in the limitations, the Child Supplement dataset’s use of the 5-item rather than the 25-item version of the SDQ instrument may be problematic. We decided to select all items in the scale in keeping with convention and to capture as much as the scale is capable of.

### 2.3. Independent Variables

Independent variables in the model included the policy context (e.g., health insurance type, well-child checkups, welfare provisions), and intermediary social determinants such as the household socioeconomic position, including problems paying medical bills.

*Policy Context:* Three items that measure access to and use of policy provisions or safety nets were used to examine the policy context of a child’s structural social determinants of health. These include *adequate insurance* to cover needed services, which is a national policy requirement to ensure child wellbeing [6]. The type of health insurance question asked, if and where available, whether the child had access to private, public (including military), or no health insurance. *Well-child checkups*. Following the Affordable Care Act of 2010, several provisions were made to address the mental health needs of children, including the availability and child’s use of policy-instituted well-visits (checkups) for screenings and assessments [31,36]. The survey asked if the children had any well-child checkups in the past 12 months. *Welfare provisions*. The question on the availability and/or use of welfare provisions (should the child’s family have the need for it), asked if any family member received income from a state or country welfare program and if any family member had received food stamp/SNAP benefits in the past year. These two items were combined to measure the policy-influenced welfare provision available to the child’s family.

*Household socioeconomic position (SES)*. An inputted family Federal Poverty Level (FPL) scale was used to measure the child’s family’s SES. The FPL is a value issued annually by the U.S. Department of Health and Human Services (DHHS) and Census Bureau to determine the poverty status and eligibility for welfare programs/benefits of a household. The FPL is calculated based on all sources of a family’s income computed from questions about the family’s wages, self-employment income, interests, social security, supplemental security income, other income sources, and the number of persons in the household. Households are designated to a poverty level ranging from 0–199% FPL (for the lower levels of poverty), 200–299% FPL, 300–399% FPL, and 400% FPL or higher (for extreme levels of poverty) [6].

*Problem paying medical bills*. We included the question on problems paying medical bills as an indicator of household socioeconomic position, where parents/caregivers provided a yes or no answer.

*Mediating variables*. Mediating variables in this model included the number of chronic diseases, child food insecurity, and health systems.

*Child food insecurity.* Child food insecurity was examined as a mediating and intermediary SDoH. The responding adult answered questions from a modified version of the Core Food Security Module (CFSM; α = 0.89) to ascertain the food problems in the family with specific reference to the child. The parent/caregiver was asked if in the past 30 days, “the child’s family often or sometimes worried that food would run out before having enough money to buy more”, and if the “child’s family’s food just did not last, and they did not have enough money to get more”. Higher values on the scale represented high food insecurity.

*Health system*. Two binary (yes/no) items, namely a child’s visit to a mental health or a medical specialist, constituted the intermediary health system determinants. Respondents were asked if the child had received care from a mental health professional such as a psychiatrist, psychologist, psychiatric nurse, or clinical social worker in the past 12 months, while the other question asked if they had seen or talked to/received care from a medical specialist in the past 12 months.

*The number of chronic health conditions*. Respondents were asked if they were ever told by health professionals that the child had any of 27 listed chronic health conditions. Responses to these questions were re-coded into three categories: children with no conditions or problems reported; children with one condition or problems reported; and children with two or more problems or chronic conditions reported.

*Overall health status*. The respondent reported the child’s overall health status, categorized as either excellent/very good, good, or fair/poor.

*Covariates.* The child’s gender, age, race/ethnicity, and age and parent educational level were included as controls. The child’s gender question asked if the child was male or female. To highlight the prevalence of symptoms by age groups, we used the CAHMI grouping of children into three categories: 4 to 5 years, 6 to 11 years old, and adolescents aged 12 to 17 years [6]. The race/ethnicity of the children was obtained from an inputted tabulation of Whites/non-Hispanics, Black/non-Hispanics, Hispanics, and non-Hispanic/multi-racial groups.

### 2.4. Statistical Analysis

**Figure 1:**
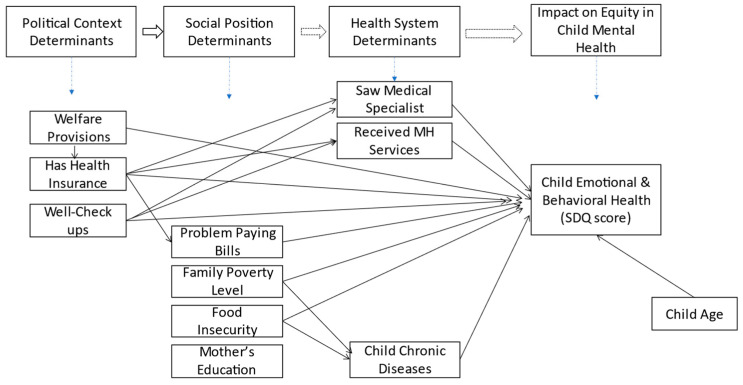
Hypothesized Path Model to Adapt the Social Determinants of Health Framework to examine Child Emotional and Behavioral Health.

This study used the above-mentioned measures to examine significant determinants, as illustrated in the adapted and hypothesized SDoH model shown in Figure 1. Statistical analysis using Stata 15.0 [37] involved data management, exploratory and confirmatory factor analysis, and path analysis/structural equation modeling (SEM) to determine the associations among SDoH constructs and child EBH problems. Descriptive characteristics of the sample were computed (see Table 1). Pearson Chi-squared estimations for various variables and the outcome (scale of child EBH as SDQ score) were also carried out.

Following the path analysis, the model fit was determined using the chi-squared test (*X^2^*), root mean error of approximation (RMSEA), comparative fit index (CFI), and the coefficient of determination (CD). The criteria for model fit included assessments of whether the *X^2^* test of model fit was statistically significant; whether RMSEA was less than 0.06; and whether CFI was greater than 0.90 [38,39]. The indirect and direct effects of the variables were also estimated. As the analytical sample was large, effect sizes low, and coefficients mostly statistically significant, most interpretations of coefficients were made based on effect size, where an effect size of at least 0.10 was considered for inclusion in the final model [40]. The maximum likelihood parameter estimation method was used for analysis, as most of the variables were normally distributed.

## 3. Results

### 3.1. Demographics

The analytical sample consisted of children (n = 9205), females (49%), drawn from across the 50 states of the United States. The racial and ethnic composition comprised 46% White/non-Hispanic; 14% Black/non-Hispanic; 29% Hispanic; and multiple ethnicities including American Indian/Asian/non-Hispanic (11%). At a 95% confidence interval, percentages and population estimates are weighted to represent the child population in the US. The age groups of the children ranged from 12–17 years (46%), 6–11 years (40%), and 4–5 years (14%). Most of the US children (83%) had a parent report of excellent/very good health status, while 15% reported good health and 2% of the children had a report of poor/fair health. Approximately 55% of the children (5489) have never been diagnosed with any of the 27 chronic health conditions. Of the rest, 23% had been diagnosed with at least one chronic condition, while 22% had been diagnosed with two or more chronic conditions.

More children (77%) resided in homes which were food secure, while 7% lived in marginally food-insecure families. Sixteen percent (16%) of the children in the sample lived in highly food insecure families. Seventy-five (75%) of the US children lived in a home where no one was receiving welfare or SNAP benefits from the government. Others (21%) lived in a home receiving some benefit, while 3% lived in a home that relied more heavily on benefits from welfare apparatuses. In addition, children in the sample (69%) resided in homes where a mother’s highest level of education was above high school, 20% lived in households where a high school diploma or GED holder was the highest level, while 11% resided in a US home where the highest educational level of the mother was less than high school. Additionally, 53% of the children had some form of private health insurance while 40% had only public insurance, including military insurance. There were still uninsured children, comprising 7% of the sample. Further, approximately 80% lived in homes where there was no problem paying medical bills, while 20% of households had problems paying their medical bills. Additionally, the children in the sample were evenly distributed (mean, 25%) across the four FPL levels (see Table 1).

### 3.2. EBH Outcomes across Child Characteristics

Using the SDQ instrument to screen for emotional and behavioral health problems among the children, 78% of the sample had low scores (0–2) that indicated no mental health difficulties, 18% of the children scored 3–5, implying that they had minor difficulties, while 4% of the children had high scores (6–10) indicating severe and definite difficulties in their emotional and behavioral health. An analysis of the Pearson chi-squared test for statistical significance indicated variations among the age groups, gender differences, chronic disease, health status, family poverty level, and their association with different levels of emotional and behavioral problems. These chi-square differences were all statistically significant (see Table 1).

Focusing on children with the highest SDQ scores (most EBH problems), more children (4.8%) in the 12- to 17-year age range had higher SDQ scores (severe and definite difficulties) than the other age groups. In addition, of the 16.6% of children in the sample living in households with high food insecurity, 8.1% had severe difficulties (high SDQ scores) including 5.2% and 3.1% of children living in marginally food insecure and food secure homes, respectively, who also had severe and definite difficulties. Of the 4% of children with higher scores on the SDQ scale (EBH problems), 10.6% had two or more chronic conditions, and 3.5% and 1.7% had one condition or no chronic condition, respectively. Further, 5.2% of the 39% of children in the sample that had only public health insurance appeared to fare worse in their emotional and behavioral health compared to severe difficulties in the 3.2% of those uninsured and 2.9% of children who had any form of private insurance. The percentage of boys (4.4%) with severe/definite emotional and behavioral problems was higher than girls (3.3%), and all these differences are statistically significant (see Table 1).

**Table 1:** Demographic Information of Study Sample Children (n = 9205).

Variables	Overall	SDQ Score (Difficulties) %			t or χ^2^
	N (%)	No	Minor	Severe	
Gender					
Male	4669 (51%)	76.8	18.8	4.4	19.4 ***
Female	4536 (49%)	80.3	16.4	3.3	
Age (yrs.)					
4–5 years	1269 (14%)	82.6	15.1	2.4	34.18 ***
6–11 years	3726 (40%)	80.0	16.4	3.6	
12–17 years	4210 (46%)	76.1	19.4	4.5	
Food Insecurity (FI)					
Food secure	7091 (77%)	81.4	15.1	3.1	218.2 ***
Marginal FI	630 (6.8%)	70.3	25.4	4.3	
High FI	1484 (16%)	66.2	26.3	7.5	
Insurance Type					
Any Private	5000 (54%)	81.6	15.5	2.9	79.7 ***
Public only/Military	3621 (39%)	74.0	20.8	5.2	
Uninsured	584 (6%)	80.8	16.0	3.2	
Family Poverty Level					
0–99% FPL	1913 (21%)	72.6	21.5	5.9	86.04 ***
100–199% FPL	2147 (23%)	77.0	18.2	4.7	
200–399% FPL	2624 (29%)	80.2	16.9	2.9	
400% FPL or above	2521 (27%)	83.6	14.9	2.5	
# Chronic Conditions					
None	54%	86.9	11.5	1.6	768.7 ***
1 condition	23%	78.6	18.2	3.4	
2 or more conditions	22%	58.1	32.0	9.9	
Overall Health Status					
Excellent/Very good	83%	81.0	16.3	2.7	255.6 ***
Good	15%	68.2	23.2	8.5	
Fair/Poor	2%	53.3	32.4	15.4	
Received MH Care					
No care	8.504	82.4	15.5	2.2	1.3 × 10^3^ ***
Received Care	731	34.1	42.8	23.1	
Mother’s Education Level					
Some high school	1527	77.8	18.1	4.1	61.7 ***
Some college	3785	75.8	19.8	4.4	
Associate Degree	1179	77.5	17.4	5.1	
Bachelor’s and more	2744	83.2	14.5	2.3	
Race/Ethnicity					
White/non-Hispanic	4279 (46%)				
Black/non-Hispanic	1243 (14%)				
Hispanic	2672 (29%)				
Multiple/non-Hispanic	1011 (11%)				

Note: *** = *p* ≤ 0.001; #—number of chronic diseases ranging from none to 2 or more

### 3.3. Correlations

The children’s SDQ measure had weak but positive correlations with most of the independent variables. For instance, child’s age (0.12) and child’s sex (0.11) had stronger correlations with SDQ score. The number of chronic diseases a child had was correlated with children seeing a mental health specialist (0.27), children seeing a medical specialist (0.21), families having problems with medical bills (0.14), and food insecurity (0.12). However, structural determinants such as the child’s ethnicity and highest level of education of adults in the family had negative but weak correlations, (−0.00 and −0.01, respectively) with SDQ scores. Food insecurity’s roots in the socio-economic situation of the child’s family was also highlighted and correlated with the child’s family’s poverty level (−0.39), child’s family’s use of welfare (0.31), the child having no insurance/public insurance (0.27), problems paying medical bills (0.26), the mother’s education (0.26), the child’s ethnicity (0.12), and chronic health conditions (0.12). All these correlations were statistically significant (see Table 2).

### 3.4. Path Coefficients

As shown in Table 3, an initial model analysis yielded significant coefficients to child SDQ scores (standardized values, β were reported here). For example, using mental health services was associated with higher SDQ or more EBH problems (0.30, *p* < 0.001). Other significant factors were the increasing age of the child (0.22, *p* < 0.001), and the child’s family’s use of welfare (−0.10, *p* < 0.05) which were all statistically significant. The hypothesized mediator, the presence of more than one chronic disease, was associated with seeing a mental health specialist (0.74, *p* < 0.001), problems paying medical bills (0.22, *p* < 0.001) and food insecurity (0.11, *p* < 0.001) while food insecurity in the family had a path coefficient of 0.10 (*p* < 0.01) to child SDQ. The use of mental health services was associated with problems paying bills (0.10, *p* < 0.001) while having no insurance or public insurance had no significant association or path to services. Standardized estimates yielded lower but statistically significant coefficients, with child age (0.11, *p* < 0.001), use of mental health services (0.10, *p* < 0.001), use of welfare (−0.01, *p* < 0.001), and food insecurity (0.04, *p* < 0.001) being notable paths to high child SDQ scores or more EBH problems.

An examination of the equation-level goodness of fit for the endogenous variables showed that the model explains only 0.02 variance in the SDQ score. The initial model failed significantly to reproduce the covariance matrix for the variables (with a chi-square (5) = 812.08, *p* < 0.001), although values such as the RMSEA were fair at 0.050. The analytical modification recommended adjustments to the structural model and a correlation of variables representing welfare benefits to chronic diseases and mental health service use to reduce the degrees of freedom/Chi-squared. The modified model had a better fit with *X^2^* (4) = 22.124, RMSEA = 0.021, 90% CI with bounds 0.013–0.03, CFI = 0.98, and a Tucker–Lewis Index of 0.93. The SRMR was fair at 0.006, and the coefficient of determination indicated that variables accounted for only 0.10 of the variation in SDQ scores.

The standardized direct effects were the same as the unstandardized results already reported, with the number of chronic diseases having no path to child SDQ scores. Further decomposition of the paths yielded indirect effects (standardized effects reported here). None of the other items in the study had a significant indirect effect (effect size up to 0.1) on child SDQ scores. For chronic diseases, there were no indirect paths to or from other items, nor effect sizes up to 0.1, although the weak paths from welfare (0.02) and problems paying bills (0.02) were statistically significant (*p* < 0.001). Further, there were no indirect effects of the socioeconomic health determinants on the child’s use of mental health services.

### 3.5. Total Effects

**Figure 2:**
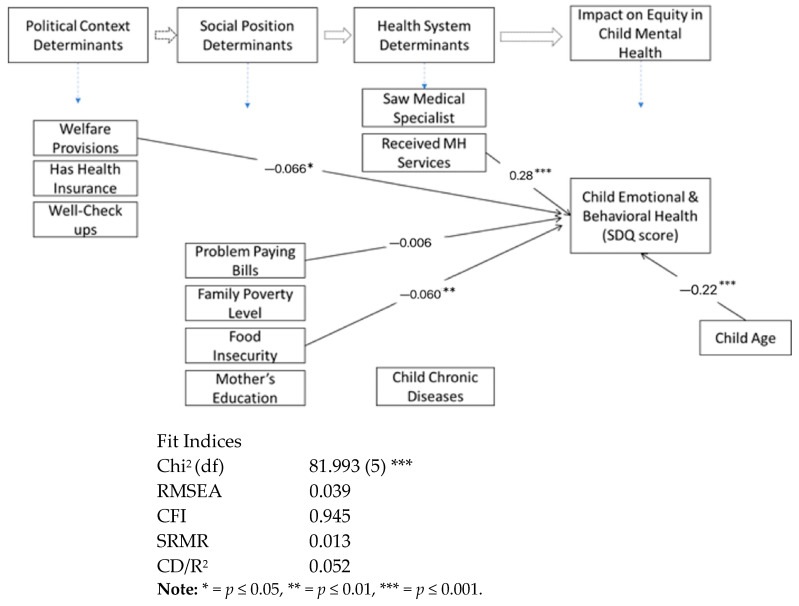
Standardized Coefficients of total effects in the final path model of social determinants of child emotional and behavioral health from the study (direct effects only).

An examination of the total effects (standardized effects reported here) highlighted the statistically significant association of the use of mental health services with child SDQ scores (0.28, *p* < 0.001) and child age (0.22, *p* < 0.001), noted in red font in Figure 2. When standardized, these total effects became 0.05 and 0.11, respectively. All total effects of paths to chronic disease were statistically significant (*p* < 0.001) except for the use of health insurance (*p* > *0*.05). Large total effects and their respective standardized values on chronic disease include the use of mental health services (0.73; 0.25), problem paying medical bills (0.26; 0.13), the use of welfare (0.15; 0.10), and food insecurity (0.10; 0.08). Likewise, the highest total effects on the use of mental health services came from the child’s family’s use of welfare provisions (0.04; 0.08), which was statistically significant at *p* < 0.001.

## 4. Discussion

The study examined the utility of constructs defined in the SDoH framework to understand how the policy, family, intermediary, and health system determinants of health are associated with child emotional and behavioral health (EBH) problems, as measured by their strengths and difficulties questionnaire (SDQ) scores. The total effects from the child’s visit to a mental health specialist and the child’s age had the highest path coefficients to the child’s emotional and behavioral health problems score. The most significant path to high levels of child emotional and behavioral health problems was for a child who had seen a mental health specialist such as a psychiatrist, psychologist, psychiatric nurse, or clinical social worker in the past 12 months. This may suggest that the existence of EBH problems drives the association and need for visits to a mental health specialist or vice versa. More likely, this recursive association could imply that households that have children with EBH problems seek mental health care, thus suggesting a proactive health-seeking behavior [25]. Further, children with higher levels of EBH problems lived in households associated with more problems paying medical bills. It is not clear whether problems with bills and more EBH problems imply that children living in lower socioeconomic contexts have more EBH problems or that healthcare expenses are challenging for the family because of the child’s EBH, an area which should be explored further in future research.

Child age was associated with the presentation of more EBH symptoms. The role of age in EBH is often ambiguous, since most EBH problems continue throughout a person’s life. Further, increasing regulation skills with age may mask the existence of EBH problems. There was no strong direct or indirect effect from the other factors on the child’s EBH, including the presence of one or more chronic diseases. However, again, factors related to the mediator (use of mental health services) such as parents having problems paying their medical bills (0.07, *p* < 0.001) and family’s use of welfare provisions (0.08, *p* < 0.001) were associated with minor effect size coefficients on child EBH, suggesting the significant indirect role of socioeconomic status on child mental health.

Respondents were asked if the child received care from a mental health professional such as a psychiatrist, psychologist, psychiatric nurse, or clinical social worker in the past 12 months or specialized medical personnel. In the initial model and in keeping with the social determinants of health theory, the non-significant association of SDQ scores with seeing specialized medical personnel underscores the often-social (non-medical) nature of factors that predict mental health outcomes (even in the presence of chronic diseases). This finding may suggest the need for a non-medical or integrated approach to health, the benefit of an SDoH framework, and how EBH challenges can benefit from focused and specialized mental health care. The strong association with visits to mental health practitioners also underscores the need for sufficient professionals such as social workers, psychiatrists, lay workers, or psychologists in every context where children can be reached and helped in large numbers, such as schools and other community centers. A multi-sector and multi-layered approach to health and social service provision can increase reach and foster the use of easily applicable and scalable interventions in child mental health.

Indeed, as argued by the proponents of the SDoH framework, the non-medical factors examined in the study are implicated in child EBH, as illustrated by their paths to significant paths. Yet, the error terms for child SDQ (0.98) were large and corroborated the coefficient of determination’s low estimate of the model’s prediction of variation in child SDQ. This low variability suggests that for children experiencing severe or more difficult problems, other factors not included in the analysis may help explain the high SDQ scores and, subsequently, EBH.

## 5. Conclusions

The study sought to answer questions such as how structural social determinants of health from the macro context, such as availability of welfare services, health insurance, and use of child well-checkups, affect a child’s EBH problems. We also examined intermediary social determinants of health, such as household socioeconomic position (poverty level) and problems paying medical bills associated with a child’s EBH problems. Further, we examined the path of the direct and indirect mediating effects of the intermediary factors such as access to mental health services on child EBH. We used the SDoH framework to model the paths of association and the extent to which a child’s sociopolitical structural determinants interact with individual and family intermediary determinants to influence outcomes in the child’s mental health. From this analysis, family socioeconomic and intermediary factors played an important role in the child SDQ scores. Individual factors such as increasing age of the child were associated with higher SDQ scores. Older children aged 12 to 17 years had higher SDQ scores (severe and definite difficulties) than the other age groups. The increased SDQ scores with age calls for more research into the underlying factors, and may underscore the importance of early identification, screening, and prevention, and the need to continue wellness checkups to monitor the mental health of young children even into their teen years. For instance, the CDC youth risk behavior survey reported that 29% of high school students experienced poor mental health or persistent feelings of sadness or hopelessness (42%) in 2021 [41].

Factors from the political/policy context were meager and not statistically significant in their effect on child SDQ scores. The strong association that seeing a mental health professional has with a child’s emotional and behavioral health calls attention to the need for more mental health providers, such as social workers, in effective catchment areas, especially the school contexts. The role of a family’s socioeconomic position and status reflected in households with problems paying medical bills, use of welfare, and the high number of children in poverty begs a review of the political context for policies, programs, and interventions that strengthen the child’s micro or family setting.

### 5.1. Limitations

We must acknowledge the limitations of the study, which include the absence of measures capturing the caregiver’s mental health, which is a critical determinant of child EBH [42,43]. Further, several EBH problems in children, including ADHD, have etiologies in genetic and biochemical factors and may account for the missing variability in predictors in this study [19,44]. However, important confounders such as chronic diseases and food insecurity were statistically controlled in the analyses, strengthening the utility of the study in examining child EBH. Further, we questioned the reliability of the 5-item SDQ as opposed to the more robust 25-item instrument for use in a nationally representative survey such as this, and the absence of other gold standard measures. Another limitation with implications for research is that the survey data were cross-sectional, so we could not explore causality. Additionally, because it is cross-sectional, it cannot be ascertained from these data if receiving mental health services reduced EBH over the long term—an important area to explore in future studies. We also acknowledge that our discussions and interpretation are based on the path analysis methodology, which is different from a multiple regression, but has utility in illustrating models that are more complex, albeit realistic. Our use of path analysis compared different models to determine which one best fits the data, and while path analysis disproves some models that hint at causality, it cannot prove causality itself.

### 5.2. Implications

This study sought to center children and their emotional and behavioral problems within the social determinants of health of the households they dwell in. In highlighting the implications of the findings, the following direct and indirect implications come to the fore. There is a need for specific and contextual policy-related interventions that reduce the EBH problems of children. Evidence across global settings offers implications for policy-related interventions that may aid children’s EBH and should be explored. The role of the socioeconomic status of families, evidenced by the associations of paths linking family poverty level, problem paying bills, use of welfare provisions, or maternal level of education, cannot be overemphasized. First, policy-related interventions that explore poverty alleviation for needy families with young children are critical to improving child EBH and access to services, and thus warrant further investigation. For example, a systematic review by Zimmerman and colleagues [45] examining the impact of cash transfers on the mental health of children and young people in middle- and low-income countries (LMIC) found evidence that 85% of cash transfer programs (e.g., both conditional and unconditional) had a significant positive impact on at least one mental health outcome among the children. Similarly, another systematic review on monetary poverty alleviation programs on children’s and adolescents’ mental health by Zaneva and colleagues [46] found small but significant reductions in adolescents’ internalizing problems following the poverty alleviation interventions compared to control groups. Cash transfer programs show promise in impacting child mental health by increasing household income and economic security, and the reduction of financial strain and stress that may heighten emotional and behavioral health risks as children grow older [45].

In addition, policy interventions that target upstream root causes that drive systematic mental health inequities are needed. These interventions may target access to education and employment opportunities, healthy food options, stable affordable housing, and safe neighborhoods that provide resources and opportunities that promote healthy child and family development, all of which are highlighted in the study, and known social determinants of mental health [47]. For example, interventions that increase the supply and affordability of housing, such as long-term rent subsidies, have shown promising mental health effects when combined with case management and other supportive services targeting complex family needs [48].

Additionally, community-level interventions, such as the redevelopment of vacant and abandoned lots and greenspace development, represent viable options for disrupting the structural conditions—such as crime and community violence—that contribute to mental health disparities [47,49]. Multicomponent community-based prevention interventions designed to enhance community partnerships and inter-organizational collaboration for improved care coordination and service delivery represent promising approaches to address multifaceted family needs. Approaches such as that of Communities that Care have demonstrated effectiveness in the areas of youth delinquency and substance use prevention [50] which may be adapted to address material hardship and youth mental and behavioral health.

This study revealed that children with EBH problems are also those who are accessing care (seeing mental health specialists); thus the need to optimize service provision as a point of intervention is critical for addressing EBH problems in children. Effective individual and family-based interventions (such as parenting skills training) [2] and practice models should be strengthened alongside poverty alleviation efforts. Furthermore, increasing access to mental health services, and the use of task-shifting strategies that empower communities to be co-creators of interventions that work, especially in settings where children can be reached easily and on time, is essential. Task- shifting processes of delegation enable, where appropriate, highly specialized mental health professionals to expand access through less specialized workers or volunteers. Some areas that require strengthening in service provision for children and families living in poverty include multidisciplinary teams, improved clinical infrastructure, effective assessment and screenings of social determinants of mental health, and care coordination, among others [51]. Finally, nationally representative child supplement data such as those used for the study need robust measures such as maternal mental health, as well as child genetic and neurobiological factors [2] that may be helpful for explaining the variation in outcomes on the child’s reported symptoms of EBH.

## Figures and Tables

**Table 2:** Correlation Coefficients of Observed Variables (n = 9205).

Variables	1	2	3	4	5	6	7	8	9	10	11	12	13
Macro/Policy factors													
1. Well Visits	1												
2. Insurance type	0.11 ^c^	1											
3. Use of welfare	−0.03 ^b^	0.34 ^c^	1										
Family SES factors													
4. Mom education	0.11 ^c^	0.43 ^c^	0.30 ^c^	1									
5. Problem med. bills	0.03 ^b^	0.17 ^c^	0.13 ^c^	0.13 ^c^	1								
6. Family Poverty	−0.76 ^c^	−0.56 ^c^	−0.52 ^c^	−0.55 ^c^	−0.20 ^c^	1							
7. Food Insecurity	0.03 ^b^	0.27 ^c^	0.29 ^c^	0.26 ^c^	0.27 ^c^	−0.39 ^c^	1						
Health System Factors													
8. Rcvd Mental care	−0.04 ^c^	0.0	0.07 ^c^	−0.03 ^a^	0.07 ^c^	−0.02	0.07 ^c^	1					
9. Rcvd Specialized care	−0.07 ^c^	−0.05 ^c^	−0.02	−0.07 ^c^	0.04 ^c^	0.06	0.01	0.14 ^c^	1				
Child Health Factors													
10. Overall Health	0.03 ^a^	0.16 ^c^	0.13 ^c^	0.18 ^c^	0.08 ^c^	−0.21 ^c^	0.17 ^c^	0.12 ^c^	0.11 ^c^	1			
11. Chronic Conditions	−0.05 ^c^	0.01	0.09 ^c^	−0.03 ^b^	0.14	−0.02	0.19 ^c^	0.26 ^c^	0.22 ^c^	0.18 ^c^	1		
Child Demographics													
12. Age	−0.10 ^c^	0.06 ^c^	0.11 ^c^	0.01	0.02 ^a^	−0.08 ^c^	−0.01	−0.07 ^c^	−0.02 ^a^	−0.03 ^b^	−0.05 ^c^	1	
13. Ethnicity	0.02 ^a^	0.23 ^c^	0.12 ^c^	0.23 ^c^	0.13	−0.25 ^c^	0.12 ^c^	−0.05 ^c^	−0.07 ^c^	0.10 ^c^	−0.07 ^c^	0.03 ^b^	1
14. Gender	0.02	0.02	0.01	0.01	0.00	−0.01	0.01	−0.04 ^c^	−0.01	−0.03 ^b^	−0.07 ^c^	0.01	0.02

Note: ^a^ = *p* ≤ 0.05, ^b^ = *p* ≤ 0.01, ^c^ = *p* ≤ 0.001.

**Table 3:** Path Coefficients and Fit Indices for Child SDQ Score (EBH) and SDOH Factors (n = 9205).

	SDQ Score (Child EBH Symptoms)			(On Figure 2)
Variables	Coeff. (S.E.)	Standardized Coeff. (S.E.)	Indirect Effects	Total Effects
Macro/Policy factors				
1. Well Visits	0.20 (0.039) ***	0.05 (0.009) ***	-	-
2. Insurance type	−0.01 (0.031)	−0.00 (0.011)	-	-
3. Use of welfare	−0.07 (0.033) *	−0.027 (0.011) *	−0.024 (−0.005) *	−0.066 (−0.024) *
Family SES factors				
4. Mom education	0.05 (0.18) **	0.03 (0.011) **	-	-
5. Problem medical bills	0.14 (0.025) ***	0.06 (0.010) ***	−0.006 (0.002)	−0.006 (0.002)
6. Family Poverty Level	−0.01 (0.021)	−0.01 (0.01)	−0.002 (−0.001)	−0.004 (−0.003)
7. Food Insecurity	0.06 (0.020) ***	0.033 (0.010) ***	−0.003 (−0.001)	0.060 (0.032) **
Health System Factors				
8. Rcvd Mental care	0.30 (0.05) ***	0.059 (0.009) ***	−0.024 (−0.005)	0.28 (0.05) ***
9. Rcvd Specialized care	0.05 (0.047)	0.01 (0.010) ***	-	-
Child Health Factors				
10. Overall Health	0.31 (0.038) ***	0.08 (0.010) ***	-	-
11. Chronic Conditions	−0.033 (0.021) ***	−0.019 (0.010) ***	-	−0.031 (−0.019)
Child Demographics				
12. Age	0.22 (0.020) ***	0.11 (0.009) **	−0.001 (−0.001)	−0.22 (0.11) ***
13. Ethnicity	−0.08 (0.015) ***	−0.05 (0.009) ***	-	-
14. Gender	−0.10 (0.031) ***	−0.03 (0.009) **	-	-
Error terms				
e.sdq score	1.98 (0.027)	0.98 (0.002)		
e.chronic diseases	0.60 (0.008)	0.90 (0.005)		
e.mental health care	0.08 (0.001)	0.99 (0.002)		
Fit Indices				
Chi^2^ (df)		81.993 (5) ***		
RMSEA		0.039		
CFI		0.945		
SRMR		0.013		
CD/R^2^		0.052		

Note: * = *p* ≤ 0.05, ** = *p* ≤ 0.01, *** = *p* ≤ 0.001.

## Data Availability

Data used for the study are from the Child and Adolescent Health Measurement Initiative (CAHMI), 2016, Data Resource Center for Child and Adolescent Health, www.childhealthdata.org (accessed on 15 March 2017).
